# Analysis of the prognostic value of thyroglobulin antibody change trends during follow-up after ^131^I treatment in patients with differentiated thyroid carcinoma

**DOI:** 10.3389/fonc.2025.1496594

**Published:** 2025-02-04

**Authors:** Hua Ge, Wenxin Chen, Zhiyi Lin, Yuxuan Li, Shuting Chen

**Affiliations:** Department of Nuclear Medicine, Shengli Clinical Medical College of Fujian Medical University, Fujian Provincial Hospital, Fuzhou University Affiliated Provincial Hospital, Fuzhou, China

**Keywords:** differentiated thyroid carcinoma, 131I, thyroglobulin antibody, prognosis, persistent/recurrent DTC

## Abstract

**Objectives:**

The prognostic value of thyroglobulin antibody (TgAb) and its trends during follow-up periods may guide the treatment plans in patients with differentiated thyroid cancer (DTC) following surgery, however, there is still a lack of sufficient data. This study aims to evaluate the impact of change trends in TgAb levels on the prognosis of patients with DTC and to explore its potential application in clinical practice.

**Materials and methods:**

A retrospective analysis was conducted on the medical records of 2,981 DTC patients who underwent surgery followed by ^131^I treatment. Among these, 248 patients with positive TgAb before treatment and had a follow-up period at least 12 months were included. Patients were categorized into four subgroups based on changes in TgAb levels: the Negative Conversion Group (TgAb shifted from positive to sustained negativity), the Decrease Group (TgAb decreased by more than 50% but remained positive), the Stable Group (TgAb fluctuated by ≤ 50% throughout follow-up), and the Increase Group (TgAb increased by 50% or more). Clinical and histopathological data among the four groups, as well as disease persistence/recurrence status after ^131^I treatment, were compared.

**Results:**

Pre-treatment TgAb levels in the Negative Conversion Group were significantly lower than those in the other three groups (*P*<0.001). Compared to the Negative Conversion Group, the Stable Group had more postoperative lymph node metastases (*P*<0.05). Although pre-treatment TgAb levels in the Increase Group were lower than those in the Decrease Group, the Increase Group required significantly more treatments and a higher total dose of ^131^I (*P*<0.05). Analysis of the relationship between TgAb trends and treatment outcomes revealed 34 cases of recurrent/persistent DTC. The Negative Conversion Group had significantly better outcomes than the Stable Group and Increase Group (*P*<0.001, *P*=0.007), while the Decrease Group showed better outcomes than the Stable Group (*P*=0.045).

**Conclusions:**

Negative conversion or a decrease in TgAb levels was associated with a favorable prognosis, whereas stable or increased TgAb levels indicated a higher risk of persistent/recurrent DTC. For patients with positive TgAb serum levels, monitoring the TgAb trend changes during follow-up should be a clinical priority, with timely adjustments to individualized treatment plans.

## 1 Introduction

Patients with differentiated thyroid carcinoma (DTC) generally have a favorable prognosis, with a 10-year survival rate exceeding 90%. However, recurrence occurs in 5% to 20% of patients, and due to its high incidence, DTC is the leading cause of mortality among endocrine tumors (1). Thyroglobulin (Tg), a thyroid-specific protein, is recognized as a specific tumor marker for the postoperative diagnosis and monitoring of persistent/recurrent disease in DTC (2). TgAb may arise as a response to the release of Tg produced by thyroid cancer cells or thyroid tissue (3). In principle, following total thyroidectomy and ^131^I treatment, TgAb levels in DTC patients should gradually decline in TgAb levels to negativity due to the absence of Tg antigen stimulation from cleared thyroid follicular cells. Clinically, about 20% to 30% of DTC patients test positive for TgAb (4, 5), which interferes with the accuracy of Tg detection and thereby limits its utility as a tumor marker during follow-up. Consequently, managing follow-up and selecting of treatment options for postoperative TgAb-positive DTC patients remain challenging.

Some researchers have suggested that serum TgAb could serve as a surrogate tumor marker for disease recurrence during long-term follow-up in patients undergoing combined ^131^I treatment after DTC surgery (6, 7). However, this hypothesis remains contentious, with no consensus on follow-up protocols or the prognostic value of TgAb positivity in DTC patients (8). Furthermore, the relatively low prevalence of TgAb positivity and the prolonged time required for seroconversion to negativity pose challenges for research. Many studies (6, 9) are limited by small sample sizes and short follow-up periods.

This study aims to evaluate the prognostic significance of TgAb change trends in postoperative TgAb-positive DTC patients after ^131^I treatment. By retrospectively analyzing a large dataset spanning over a decade and comparing clinical, histopathological, and treatment outcomes across different TgAb trajectories, this study seeks to enhance the clinical applicability of TgAb as an alternative tumor marker.

## 2 Methods

### 2.1 Study population

A retrospective analysis was performed on 2,981 DTC patients who underwent ^131^I treatment between January 2012 and September 2023, patients with positive TgAb before ^131^I treatment were included in the study if they met all of the following criteria. The inclusion criteria were 1) patients underwent total thyroidectomy with a confirmed pathological diagnosis and complete postoperative clinical data, 2) the first ^131^I ablation treatment was performed in our department, 3) serum TgAb levels were positive (≥115 IU/mL) before the initial ^131^I treatment, with subsequent measurements conducted using standardized equipment and assay methods, 4) regular follow-up for at least 12 months after ^131^I treatment, with complete relevant serological and imaging examinations, and 5) no history of medications that could interfere with TgAb levels. The exclusion criteria were 1) postoperative pathology revealed other thyroid cancer types or DTC combined with other thyroid cancers, 2) presence of other malignant tumors or severe hepatic or renal insufficiency, as well as cardiovascular system diseases, or 3) lack of standardized treatment and regular follow-up in our hospital led to incomplete relevant information. This study adhered to the principles of the Declaration of Helsinki, and all patients provided written informed consent.

### 2.2 ^131^I treatment and follow-up

^131^I treatment protocols were implemented in accordance with the Guidelines for ^131^I Treatment of Differentiated Thyroid Carcinoma. Patients discontinued levothyroxine and followed a low-iodine diet for 3-4 weeks before ^131^I therapy. Pre-treatment evaluations included measurements of Tg, TgAb, and TSH levels, along with complete blood counts and liver and kidney function tests. Imaging studies, such as neck ultrasound, chest CT, and thyroid static imaging, were routinely performed. For suspected distant metastasis, additional diagnostics, including Dx-WBS with ^131^I, ^18^F-FDG PET/CT, or local CT/MRI, were conducted. Post-treatment whole-body scans (Rx-WBS) were performed 4-7 days after ^131^I therapy. The first outpatient follow-up occurred two months post-treatment, during which levothyroxine doses were adjusted based on test results. Subsequent follow-ups were scheduled every 3-6 months and included thyroid function tests and/or neck ultrasound. Additional imaging studies were performed as needed to monitor distant metastases. In cases of suboptimal treatment outcomes, repeat ^131^I treatment was administered at 6-12 month intervals as necessary.

### 2.3 Laboratory measurement

Serum levels of TSH, Tg, and TgAb were measured using electrochemiluminescence immunoassay (ECLIA) on the cobas 6000 analyzer (Roche Diagnostics, Germany). The measurement range for TgAb was 10-4000 IU/mL. Due to the heterogeneity of autoantibodies, which could lead to non-linear sparse phenomena, samples exceeding the upper limit of detection were not diluted. For this study, TgAb levels ≥4000 IU/mL were recorded as 4000 IU/mL.

### 2.4 Imaging examination

Rx-WBS was performed 4-7 days following oral administration of the therapeutic dose of ^131^I, while Dx-WBS was conducted 48 hours after ingestion of the diagnostic dose of ^131^I. A Discovery 670 dual-head SPECT/CT imaging system (GE Healthcare) was used, equipped with high-energy parallel hole collimators. Data acquisition was performed at a scanning speed of 9.2-10 cm per minute. Routine neck fusion tomography was conducted, with additional fusion imaging of suspicious areas when necessary.

### 2.5 Assessment of persistent/recurrent DTC post-^131^I treatment

The presence of persistent/recurrent DTC after ^131^I treatment was determined by integrating imaging results, including ^131^I-WBS, neck ultrasound, and chest CT, with cytological or histological examinations where applicable. According to the Consensus on the Diagnosis and Treatment of Recurrent Metastatic DTC by the Thyroid Cancer Expert Committee of the Chinese Society of Clinical Oncology, persistent/recurrent DTC is defined as the detection of new lesions or tumor remnants during follow-up after treatments such as surgery, thyroid clearance, and/or TSH suppression (10). Recurrence or residual tumors could occur in the thyroid bed or extrathyroidal sites via lymphatic, hematogenous, or implantation routes.

### 2.6 Observational indicators

The observational indicators included gender, age, primary tumor size, number of lymph node metastases, TNM stage, coexistence with Hashimoto’s thyroiditis (HT), pre-^131^I treatment TgAb levels, times of radioiodine, cumulative dose of radioiodine, and tumor persistence/recurrence status. Tumor TNM stage was conducted following the 8th edition of the AJCC Cancer Staging Manual by the American Joint Committee on Cancer and the International Union Against Cancer (11).

### 2.7 Grouping

Patients were categorized based on change trends in TgAb levels at the final follow-up, using definitions from existing literatures (6, 12): Negative Conversion Group: TgAb shifted from positive to sustained negativity(negativity defined as <115 IU/mL). Decrease Group: TgAb levels decreased by more than 50% but remained positive. Stable Group: TgAb levels fluctuated by ≤50% (increase or decrease) throughout the follow-up period. Increase Group: TgAb levels increased by more than 50%.

### 2.8 Statistical methods

Data were compiled into an Excel database and analyzed using SPSS 26.0 software. Quantitative data with a normal distribution were expressed as the Mean ± SD, and intergroup comparisons were performed using one-way ANOVA. Quantitative data that did not conform to a normal distribution were reported as medians (Q1, Q3), with intergroup comparisons made using the Kruskal-Wallis H test. Categorical data were expressed as percentages (%), and intergroup comparisons were made using the Pearson chi-square test or Fisher’s exact test. Kaplan-Meier survival analysis was employed to evaluate the relationship between the groups and disease persistence/recurrence. And the predictive value of relevant clinical factors for disease persistence/recurrence was analyzed using the Receiver Operating Characteristic (ROC) curve. Statistical significance was defined as *P*<0.05.

## 3 Results

### 3.1 Group distribution

**Figure 1:**
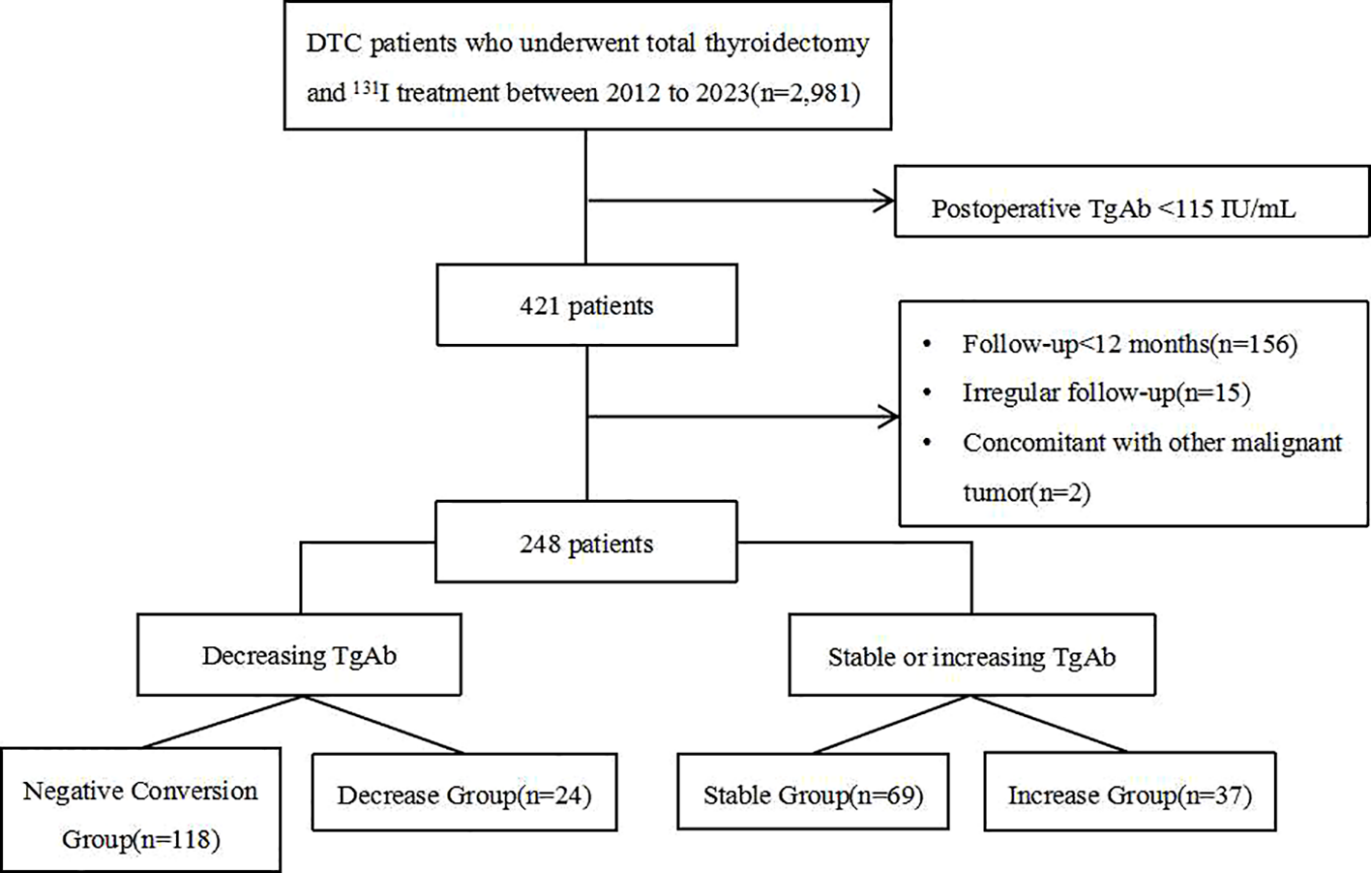
Flow chart of the study population and results.

A total of 248 eligible DTC patients with positive TgAb were included in this study (**Figure 1**). The cohort comprised 42 males and 206 females with an average age of 41.7 ± 13.0 years. Among them, there were 242 cases of papillary thyroid carcinoma, 5 cases of follicular thyroid carcinoma, and 1 case of mixed thyroid carcinoma. The distribution across the groups was as follows: the Negative Conversion Group (118 cases, 47.6%), Decrease Group (24 cases, 9.7%), Stable Group (69 cases, 27.8%), and Increase Group (37 cases, 14.9%).

### 3.2 Comparison of clinical and histopathological data among different groups

As shown in **Table 1**, the pre-treatment TgAb levels differed significantly between the Negative Conversion Group and the other three groups (*P*<0.05). A statistically significant difference was observed in the number of lymph node metastases when comparing the Stable Group with the Negative Conversion Group (*P*<0.05). Significant differences were also found in pre-treatment TgAb levels, times of radioiodine, and cumulative dose of radioiodine between the Increase Group and Decrease Group (*P*<0.05). No statistically significant differences were observed in gender, age, primary tumor size, TNM stage, or coexistence with HT among the four groups (all *P*>0.05).

**Table 1:** Comparison of clinical and histopathological data in different groups.

Parameters	Negative Conversion Group(n=118)	Decrease Group (n=24)	Stable Group (n=69)	Increase Group(n=37)	*P*
Gender (n, %)					0.550
Male	19 (16.1%)	2 (8.3%)	15 (21.7%)	6 (16.2%)	
Female	99 (83.9%)	22 (91.7%)	54 (78.3%)	31 (83.8%)	
Age [years, M(Q1, Q3)]	39.5 (31.0, 48.2)	37.0 (25.2, 53.8)	44.0 (33.0, 52.0)	40.0 (32.0, 51.5)	0.241
Primary tumor size [cm, M(Q1, Q3)]	1.4 (0.9, 2.0)	1.5 (1.2, 2.2)	1.5 (1.1, 2.5)	1.2 (0.7, 2.6)	0.298
Number of LN metastases [n, M(Q1, Q3)]	6 (2, 13)	5 (2, 14)	10 (5, 18)^*^	7 (3, 21)	0.007
TNM stage (n, %)					0.059
Stage I	100 (84.7)	19 (79.2)	49 (71.0)	26 (70.3)	
Stage II-IV	18 (15.3)	5 (20.8)	20 (29.0)	11 (29.7)	
Coexistence with HT [n, %]					0.751
Yes	95 (80.5)	20 (83.3)	57 (82.6)	29 (78.4)	
No	23 (19.5)	4 (16.7)	12 (17.4)	8 (21.6)	
Pre-treatment TgAb levels [IU/mL, [M(Q1, Q3)]	254.0 (154.1, 517.8)	926.4 (663.4, 2628.8)^*^	719.9 (376.0, 2154.5)^*^	384.6 (233.1, 1082.5)^*^,^#^	<0.001
Times of radioiodine [n, M(Q1, Q3)]	1 (1, 2)	1 (1, 2)	1 (1, 2)	2 (1, 2)^#^	0.011
Cumulative dose of radioiodine [mCi, M(Q1, Q3)]	100 (100, 200)	100 (100, 200)	150 (100, 200)	200 (100, 250)^#^	0.015

*P<0.05 compared to the Negative Conversion Group (*H*=11.987, -2.715, -6.209, -5.478; *P*=0.007, 0.04, <0.001, <0.001 respectively).

^#^P<0.05 compared to the Decrease Group (*H*=2.810, -3.210, -2.692; *P*=0.030, 0.008, 0.043 respectively).

### 3.3 Analysis of the relationship between TgAb change trends and persistent/recurrent DTC

**Figure 2:**
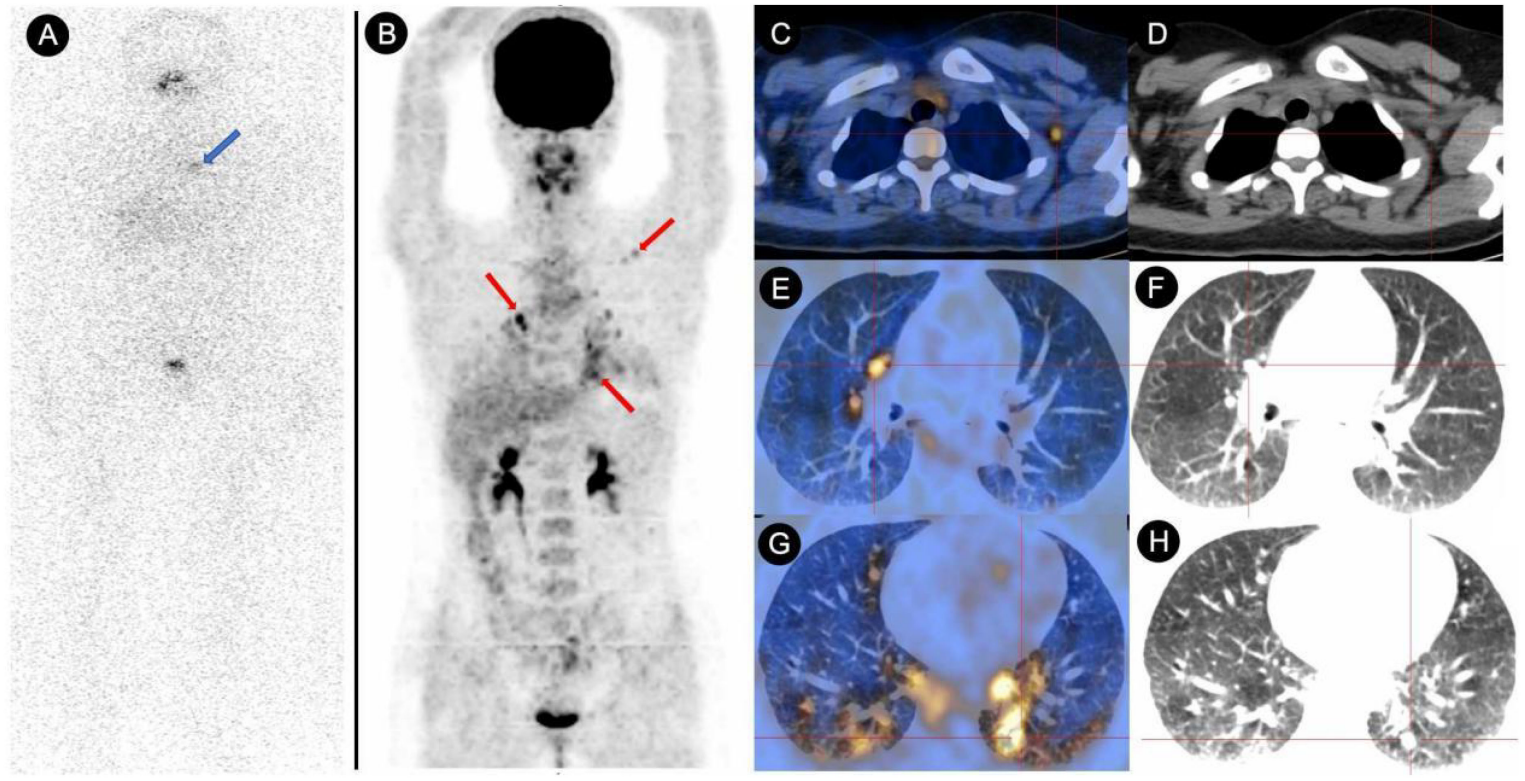
Comparative analysis of ^131^I-WBS and ^18^F-FDG PET/CT in a DTC patient with persistently elevated TgAb (>4000 IU/mL) over a 48-month follow-up after ^131^I treatment. **(A)** The 131I-WBS revealed no uptake of ^131^I in any lesions throughout the body (the area indicated by the blue arrow showed abnormal accumulation of ^131^I, which was confirmed by SPECT/CT to be physiological uptake by the thymus). **(B–H)** The whole-body MIP (Maximum Intensity Projection) image from the ^18^F-FDG PET/CT scan, as well as the fused PET/CT and CT images of metastatic lesions, indicating multiple lymph node metastases and lung metastases (red arrow and cross).

**Figure 3:**
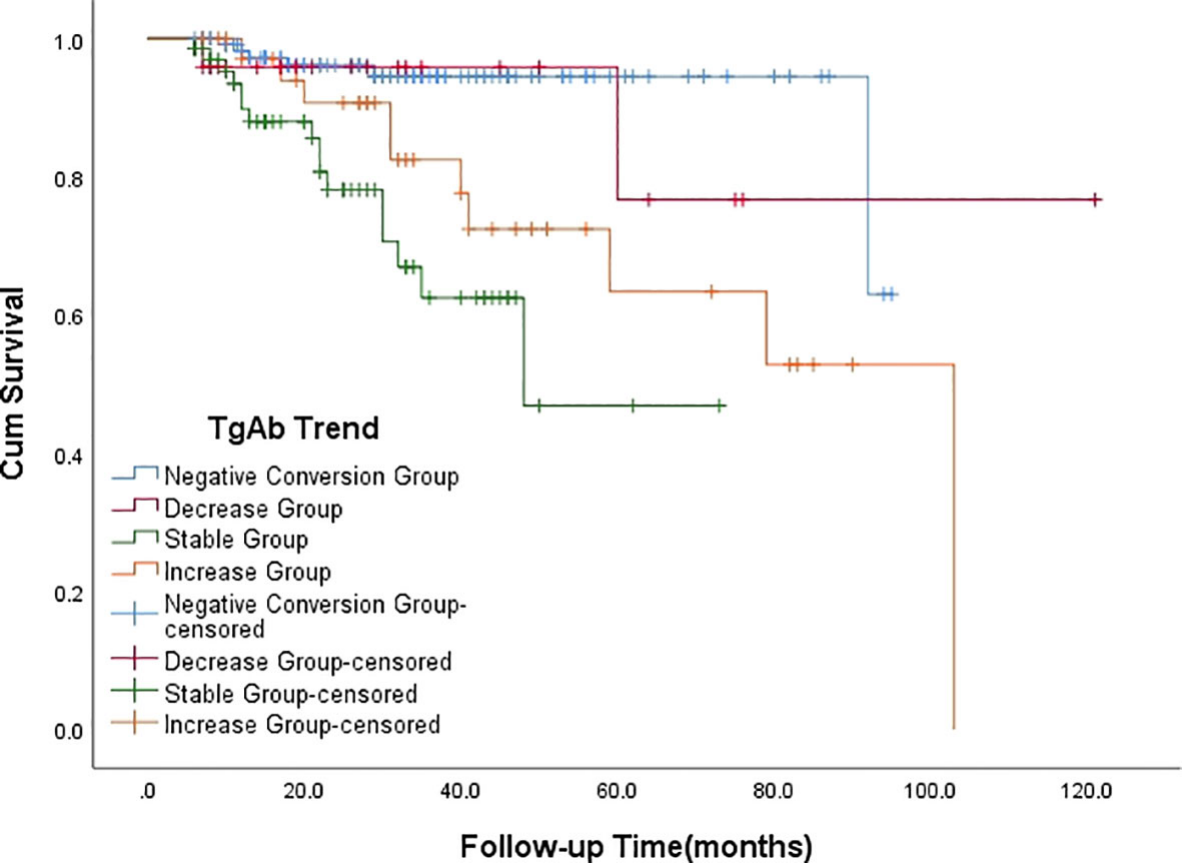
Kaplan-Meier survival analysis curve depicting the relationship between the outcome of TgAb and the persistence/recurrence of disease.

Following ^131^I treatment, 34 patients had persistent/recurrent disease at the final follow-up. Breakdown by group: Negative Conversion Group, 6 cases; Decrease Group, 2 cases;Stable Group, 16 cases; Increase Group, 10 cases. Seven patients were confirmed to have radioiodine refractory-differentiated thyroid cancer (RAIR-DTC), including 1 case in the Negative Conversion Group, 4 cases in the Stable Group (**Figure 2**), and 2 cases in the Increase Group. Kaplan-Meier survival analysis curves (**Figure 3**) demonstrated statistically significant differences in disease persistence/recurrence among the groups. Specifically, the Negative Conversion Group exhibited a significantly lower rate of disease persistence/recurrence compared to both the Stable Group (X²=19.830, *P*<0.001) and Increase Group(X²=7.373, *P*=0.007). Additionally, the Stable Group had a higher rate than the Decrease Group (X²=4.014, *P*=0.045).

### 3.4 Analysis of the relationship between relevant initial clinical factors and persistent/recurrent DTC

**Figure 4:**
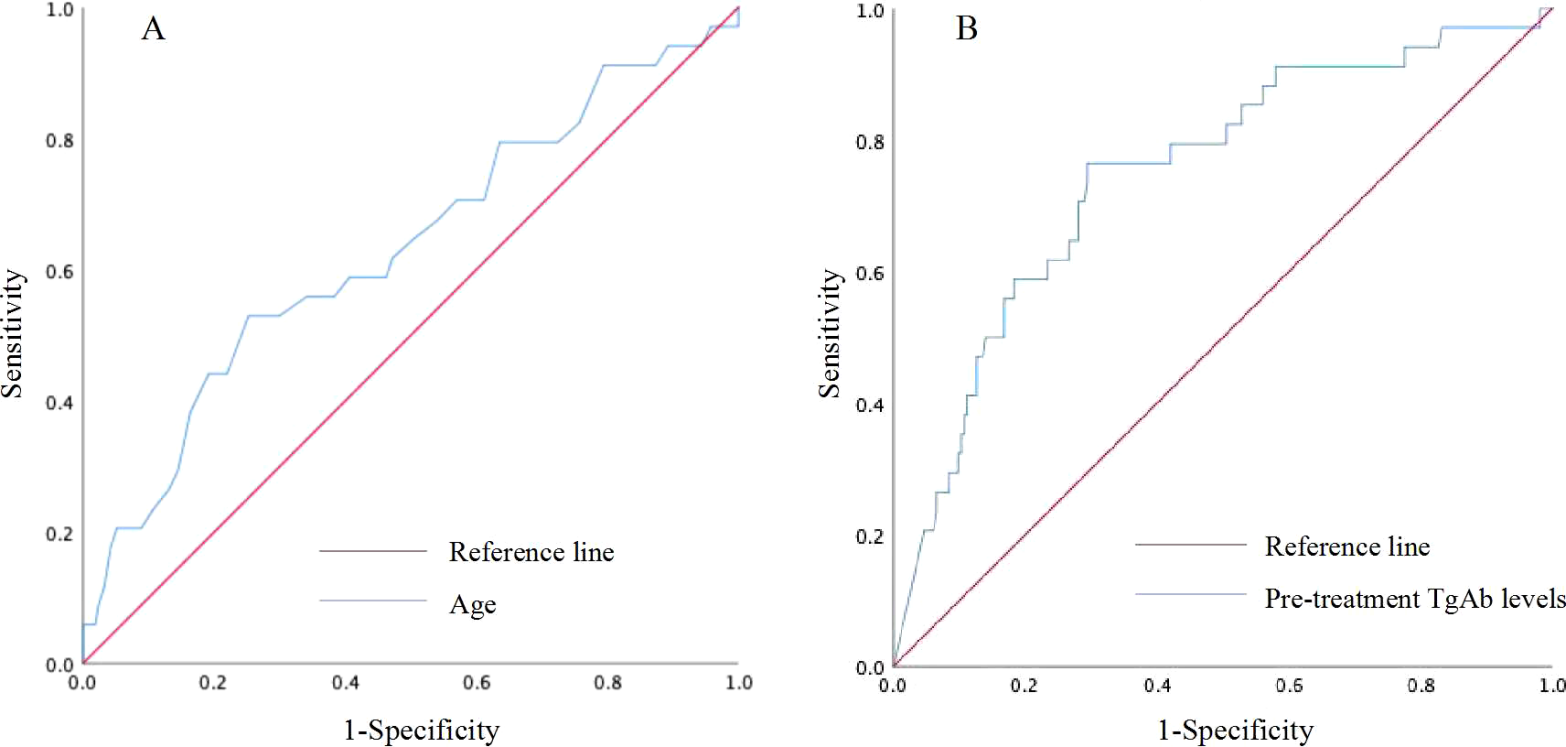
Receiver operating characteristic (ROC) curve for predicting persistent/recurrent DTC based on age **(A)** and pre-treatment TgAb levels **(B)**.

No statistically significant differences were observed in gender, primary tumor size, number of lymph node metastases, or coexistence with HT between patients with persistent/recurrent DTC and those without (all *P*>0.05). However, patients with persistent/recurrent DTC were significantly older, had higher level of pre-treatment TgAb levels, and were at a higher TNM stage compared to those without (all *P*<0.05) (**Table 2**). The age threshold predictive of persistent/recurrent DTC was 48.5 years, with an area under the ROC curve (AUC) of 0.630 (95% CI: 0.522-0.739; *P*=0.015), yielding a sensitivity and specificity of 52.9% and 74.8%, respectively. Regarding pre-treatment TgAb levels, the optimal cutoff for predicting persistent/recurrent DTC was 659.75 IU/mL, achieving an AUC of 0.749 (95% CI: 0.659-0.839; *P*<0.001), with sensitivity and specificity for this prediction were 76.5% and 70.6%, respectively (**Figure 4**).

**Table 2:** Comparison of clinical and histopathological data before the first ^131^I treatment mong patients with different treatment outcomes.

Parameters	Persistent/recurrent DTC (n=34)	Non-persistent/recurrent DTC (n=214)	*P*
Gender (n, %)			0.905
Male	6 (17.6)	36 (16.8)	
Female	28 (82.4)	178 (83.2)	
Age [years, M(Q1, Q3)]	49 (36, 55.8)	40 (31,48.8)	0.008
Primary tumor size [cm, M(Q1, Q3)]	1.6 (1.1, 3)	1.5 (1.0, 2.1)	0.222
Number of LN metastases [n, M(Q1, Q3)]	16 (5, 22)	8 (4, 15)	0.211
TNM stage (n, %)			*<0.001*
Stage I	13(38.2)	185(86.4)	
Stage II-IV	21(61.8)	29(13.6)	
Coexistence with HT [n, %]			0.793
Yes	7 (20.6)	40 (18.7)	
No	27 (79.4)	174 (81.3)	
Pre-treatment TgAb levels [IU/mL, M(Q1, Q3)]	1027.35 (679.15, 3375)	402.1 (220.76, 925.95)	*<0.001*

## 4 Discussion

By analyzing the trends of TgAb levels during follow-up after ^131^I treatment in DTC patients, this study has revealed a significant correlation between these changes and the risk of disease persistence/recurrence. These findings not only provides a new perspective for the prognostic assessment of DTC patients but also lays the foundation for the development of individualized treatment strategies. Specifically, the dynamic changes in TgAb levels can offer clinical physicians more precise indicators for disease monitoring, enabling the early identification of patients at high risk for disease progression. This, in turn, allows for timely adjustments to treatment plans, with the aim of achieving better therapeutic outcomes.

The secretion of Tg antigen by residual thyroid tissue or DTC lesions is a necessary condition for the continuous production of TgAb. TgAb levels can reflect the characteristics of the primary DTC lesion to some extent, and high levels of TgAb may indicate a longer tumor size, multiple lesions, and a higher probability of capsular invasion and extrathyroidal extension (13–15). Even though some scholars suggest that the presence of TgAb lacks predictive value for the persistent/recurrent DTC, suggesting that biochemical prediction of recurrence or persistence is not always possible (9).The majority of opinions still indicate that in patients treated with ^131^I, pre-treatment TgAb levels can be used to estimate disease risk (16), moreover, sequential changes in TgAb levels after treatment are effective predictors of disease prognosis and contribute to clinical decision-making (1, 6, 17). A research by Ernaga-Lorea A et al. (13) indicates that a 1% higher decrease in TgAb levels leads to a 1.6% decrease in the risk of persistence/recurrence during the follow-up. Our study demonstrates that approximately 57.3% of TgAb-positive patients experienced a gradual decrease in TgAb levels after ^131^I treatment, with the majority achieving a negative conversion over an extended follow-up period. The incidence of disease persistence/recurrence was lower and the prognosis was better in the Negative Conversion Group compared to both the Stable Group and the Increase Group, as well as in the Decrease Group compared to the Stable Group, aligning with the results of most studies (18, 19). For these patients, serial assessments of highly sensitive Tg and TgAb may suffice for monitoring.

After total thyroidectomy and ^131^I treatment, an increasing TgAb level over time is associated with a higher risk of structural disease and may predict tumor recurrence and metastasis (12, 19, 20). This study revealed that patients in the Increase Group showing worse prognostic outcomes and requiring more frequent and higher doses of radioiodine compared to the Decrease Group. Therefore, in patients with rising TgAb levels, heightened surveillance is necessary, incorporating a combination of serological and imaging diagnostic procedures.

The greatest controversy has been the significance of a stable TgAb level over time for disease prognosis in recent years. A study by de Meer et al. (6) found no correlation between persistent TgAb and higher recurrence or mortality rates. However, our study shows that approximately 23.2% of patients in the Stable Group experienced persistent/recurrent DTC, significantly higher than the Decrease Group. The study by Sun D et al. (20) also indicates that patients with long-term stable TgAb levels appear to have a higher risk of developing structural disease compared to those whose TgAb levels significantly decrease. This discrepancy may be related to differences in sample size and the comprehensiveness of imaging examinations between studies. For example, our study found that a considerable proportion of patients with persistent/recurrent disease in the Stable Group had dedifferentiated iodine-refractory DTC, making the lesions more elusive and prone to being overlooked. Clinically, there is a tendency to focus more on patients with rising TgAb levels and conduct further examinations, while those with stable levels may not receive the same attention. This could lead to delays in diagnosis and disease progression in these patients.

This study also revealed that patients in the Stable Group had more postoperative lymph node metastases than those in Negative Conversion Group. The number of lymph node metastases reflects tumor invasiveness and metastatic potential, and an increase in this number is generally associated with disease progression and poorer prognosis (21, 22), approximately 15% of thyroid cancer patients with regional lymph node metastases experience regional invasion, distant metastasis, and therapeutic resistance (23). The mechanism by which TgAb contributes to lymph node metastasis of DTC is not yet fully understood. The underlying reason for higher levels of TgAb in DTC patients with lymph node metastasis may be that advanced tumors can elicit a stronger immune response within the thyroid or lymph nodes, leading to enhanced expression of Tg or an increased capacity to induce the production of TgAb (24). Therefore, patients exhibiting consistently high and stable serum TgAb levels should be managed in a manner similarly to those with elevated TgAb levels. It is advisable to consider additional structural, functional, or hybrid imaging examinations during their follow-up care (25). Especially when there are suspect lesions but the ^131^I-WBS is negative, further complementary examinations such as ^18^F-FDG PET/CT and histopathology should be considered.

Our study demonstrate that TgAb levels before initial ^131^I treatment is an important potential predictor for prognosis of DTC patients, the pre-treatment TgAb levels in the Negative Conversion Group is significantly lower than the other groups. This finding aligns with previous research, which suggests that lower TgAb levels are associated with better treatment responses and outcomes (26). However, changes of TgAb levels may lag behind the progression of the disease and can be influenced by a multitude of factors, including the patient’s immune system, the immunogenicity of Tg, and the development of the tumor. We observed that the Decrease Group had the highest pre-treatment TgAb levels, yet did not exhibit adverse prognostic outcomes. For those patients, one possible explanation is that a relatively high level of TgAb may require a longer time to achieve negative conversion. Therefore, for patients who enter the follow-up period after treatment, the trend of TgAb changes is more informative for monitoring prognosis than its absolute value, a perspective also proposed by Bueno et al. (27).

The main strengths of our study include the strict follow-up for a median duration of nearly three years, standardized diagnosis and treatment processes at a large tertiary hospital, and the homogeneity of the DTC patient samples from the same geographical area in southeast China over more than a decade. While the data provided are not novel, they contribute to the scientific understanding of a clinically significant topic. However, this study also has several limitations. Firstly, the single-center, retrospective design may introduce selection bias. Secondly, the long time span, variations in technical standards, incomplete clinical parameters such as genetic testing and immunological analysis, and varying degrees of detail in postoperative pathological descriptions have led to a considerable amount of data being excluded from the study.

In conclusion, when TgAb is positive before the initial ^131^I treatment, the change trends of TgAb during follow-up should be a critical focus in clinical practice. Particularly for patients over 48.5 years old, with pre-treatment TgAb levels ≥ 659.75 IU/mL or with a higher TNM stage that associated with an increased risk of persistent/recurrent DTC. Negative conversion or a decrease in TgAb levels signifies a good prognosis, while stable or increased TgAb levels indicate a higher risk of tumor persistence or recurrence.

## Data Availability

The original contributions presented in the study are included in the article/Supplementary Material. Further inquiries can be directed to the corresponding author.
